# Birth order and health events attributable to alcohol and narcotics in midlife: A 25-year follow-up of a national Swedish birth cohort and their siblings

**DOI:** 10.1016/j.ssmph.2022.101219

**Published:** 2022-08-28

**Authors:** Lauren Bishop, Kieron Barclay

**Affiliations:** a Department of Public Health Sciences, Stockholm University, 106 91, Stockholm, Sweden; b International Max Planck Research School for Population, Health and Data Science, Max Planck Institute for Demographic Research, 18057, Rostock, Germany; c Swedish Collegium for Advanced Study, 752 38, Uppsala, Sweden; d Department of Sociology, Stockholm University, 106 91, Stockholm, Sweden; e Max Planck Institute for Demographic Research, 18057, Rostock, Germany

**Keywords:** Alcohol, Birth order, Hospitalization, Midlife, Narcotics, Sweden

## Abstract

Higher birth order is associated with increased risks of adverse health outcomes attributable to alcohol or narcotics in adolescence, but it remains unclear whether these observed birth order effects are also present in midlife. Drawing on a national Swedish cohort born in 1953 and their siblings, we estimate associations between birth order and alcohol- or narcotics-attributable hospitalization or death with a 25-year follow-up to assess whether birth order differences are observed during this life course period. Health events attributable to alcohol or narcotics use were identified using the Swedish National Patient and Cause of Death registers, respectively. We apply Cox proportional hazards models to estimate average birth order differences in hazards for alcohol- or narcotics-attributable hospitalization or death between ages 30 and 55. We estimate birth order differences between families, and use two fixed-effects approaches to estimate birth order differences within families and within families of the same type. Bivariate results indicate increased hazards for both outcomes with higher birth order; however, these results are no longer observed after adjustment for familial background characteristics in all models. Our results thereby show limited evidence for birth order differences in midlife. This study highlights that shared factors within the family of origin may be stronger predictors of adverse health outcomes attributable to substance use among siblings during this life course period. Future research should disentangle the contributions of the social environment within the family of origin for adverse health outcomes attributable to alcohol or narcotics among siblings.

## Highlights

• We estimate birth order differences for alcohol or narcotics use outcomes between ages 30-55.
• Birth order differences are not observed for alcohol- or narcotics-attributable events in midlife.
• Family background characteristics may be stronger predictors of these outcomes.

## 1 Introduction

Previous studies suggest that birth order within the family of origin may predict later health, and typically find higher risks for adverse mental and behavioral health outcomes among later-born children compared to their earlier-born counterparts. In particular, prior research indicates that higher birth order is associated with higher likelihood of suicide attempts (Easey et al., 2019), psychiatric morbidity (Riordan et al., 2012), and mortality due to suicide, accidents, and events of undetermined intent (Barclay & Kolk, 2015; Bjørngaard et al., 2013; Rostila et al., 2014). Importantly, compared to first-borns, higher birth order is associated with a higher likelihood of using alcohol and narcotics (Argys et al., 2006) and hospitalization for alcohol or narcotics use before reaching young adulthood (Barclay et al., 2016; de Veld et al., 2018). However, the extent to which birth order contributes to differential alcohol- or narcotics-attributable health outcomes between siblings later in the life course remains unknown.

The strong birth order effects observed during the teenage years aligns with previous scholarship on alcohol and narcotics use among siblings during this period in the life course. Evidence suggests that use of alcohol or narcotics among earlier-born siblings is predictive of use among later-borns (Kendler et al., 2013; Kothari et al., 2014). The association has been attributed to later-born siblings’ exposure and access to these substances via their earlier-born siblings (Blane & Barry, 1973; Samek et al., 2015), and has been found to persist until young adulthood (Samek et al., 2018), coinciding with the life course period during which rates of substance use tend to peak (Vasilenko et al., 2017).

Modeling of earlier-born siblings’ substance use is associated with a developmentally premature initiation of use among their later-born counterparts (Needle et al., 1986). In turn, a younger age at initiation is strongly correlated with a greater risk for ongoing use later in the life course (Hingson et al., 2006; Merikangas & McClair, 2012; Rioux et al., 2018). Birth order, reflected in the inter-sibling transmission of substance use behaviors, has implications for the initiation and use of alcohol and narcotics among later-born siblings during adolescence and into young adulthood, but it is not yet clear whether these observed birth order differences persist into midlife (here, ages 30–55), thereby yielding long-term consequences for later-born siblings.

The unilateral influence of earlier-born siblings’ substance use behavior on their later-born counterparts may decrease as individuals transition into adult social roles. The observed transmission of alcohol or narcotics use between earlier- and later-born siblings occurs when offspring are ostensibly living in the parental home. The transition to adulthood is typically characterized by such demographic transitions as entry into marriage or childbearing; these transitions are also associated with a decline in substance use (Bachman et al., 2014; Chilcoat & Breslau, 1996; Staff et al., 2010). Sources of influence resulting from the transitions to marriage or childbearing may further foster or decrease risk of hospitalization attributable to use of alcohol or narcotics (Fergusson et al., 2012; Torvik et al., 2013) beyond that of siblings. Previous research further suggests that the positive relationship between substance use-attributable hospitalization and birth order is less apparent in young adulthood (ages 20–26) compared to adolescence (Barclay et al., 2016), and birth order differences in harmful alcohol consumption in adulthood are limited (Black et al., 2016), thereby rendering the implications of birth order for substance use in midlife as yet unclear.

We use a unique, high-quality Swedish data register to ascertain birth order differences in hospitalization or death attributable to alcohol or narcotics within a cohort of individuals born in 1953 and their siblings. All children in each family were followed from ages 30–55, from the onset of adulthood through middle age, allowing us to harmonize the follow-up data on alcohol- and narcotics-attributable hospitalization or death for 25 years. Building on previous research that found strong birth order effects before reaching young adulthood, we estimate the extent to which birth order is associated with alcohol- or narcotics-attributable events in midlife to discern whether birth order differences for these outcomes are observed during this discrete life course period.

## 2 Materials and methods

### 2.1 Data and ethical approval

The data were derived from the RELINK53 data material. Created in 2017/2018, RELINK53 is comprised of a birth cohort of individuals born in 1953 and living in Sweden in 1960, 1965, and/or 1968. The register also includes the index individuals’ antecedent, contemporaneous, and descendant family members, the linkages for which were obtained from the Swedish Multigenerational Register (Almquist et al., 2020). The Stockholm Regional Ethical Review Board granted ethical permission for RELINK53 (reg no. 2017/34–31/5).

### 2.2 Study population

The study population consisted of the index individuals (hereafter, egos; n = 108,562) from the 1953 birth cohort and their linked biological siblings (n = 220,009), comprising 107,462 unique families. Linkages to the egos' siblings were ascertained via the biological mother's assigned identification number. Each mother's full fertility history was included to determine the size of each respective sibling group of which the ego is a part; however, siblings' inclusion in RELINK53 was conditional upon their survival until 1960 (Statistics Sweden, 2017).

The analytical sample was first restricted to families with two or more children, all of whom were born between 1943 and 1960 (n = 60,954 families; 168,240 individuals). We subsequently excluded families of more than five children (1420 families; 9296 individuals) as such large families are relatively uncommon in Sweden; these families comprised only 5.5% of the total sample. We further excluded families with monozygotic or dizygotic twins (1406 families; 4972 individuals) due to the lack of clarity with which birth order could be assigned. Finally, we excluded individuals who died or emigrated from Sweden prior to the start of the observation period (n = 6020). These criteria yielded a final analytical sample of 57,575 families (n = 147,952 individuals).

### 2.3 Variables

Hospitalization and death attributable to use of alcohol or narcotics were identified using records from the National Patient and Cause of Death registers, respectively. The 25-year measurement period started in 1973 for the oldest cohort and in 1990 for the youngest. The hospitalization variables were each operationalized as an inpatient event with an underlying or secondary diagnosis attributable to use of alcohol or narcotics, respectively, and included the following codes from the tenth revision of the International Classification of Diseases (ICD-10): E244; F10–16; F18–19; G312; G621; G721; I426; K292; K70; K852; K860; O354; O355; T40; T423–424; T426; T436; T51; Y90–91; Z502–3; Z714–15; Z721–22 (World Health Organization, 2019). An external review and validation of the National Patient Register showed that the positive predictive value of diagnoses is 85–95% (Ludvigsson et al., 2011).

The cause-specific mortality variables were each operationalized as a primary or underlying cause of death attributable to use of alcohol or narcotics, respectively, in accordance with the European Monitoring Centre for Drugs and Drug Addiction (EMCDDA) guidelines. The measures included the above ICD-10 codes, as well as the external cause codes for alcohol (X45; X65; Y15) and narcotics (R780; X41–42; X44; X61–62; X64; Y11–12; Y14) (European Monitoring Centre for Drugs and Drug Addiction, 2010; World Health Organization, 2019).

Conversion tables available from the Swedish National Board of Health and Welfare were used to translate codes between the ICD-10 and the eighth (1972–1986; Socialstyrelsen, 2018) and ninth (1987–1996; Socialstyrelsen, 2018a) revisions for hospitalization events and deaths occurring prior to 1997.

Birth order was included in all analyses as the main exposure, and was assigned stepwise within each respective sibling group from first-born to nth-born according to the siblings’ birth dates. The variable was subsequently coded categorically as first-through fifth-born.

There are several logical dependencies between family background characteristics that must be considered when estimating the relationship between birth order and health outcomes so that trends in these familial variables are not spuriously attributed to birth order (Black et al., 2018). We included five potential confounders to balance the effects of family background by birth order, thereby accounting for the between-family variation in birth order for alcohol- or narcotics-attributable events. The child's calendar year at birth (birth year) was included as a covariate to account for potential cohort trends in alcohol and narcotics use, and rates of hospitalization or death attributable to use, during our observation period. Birth year was applied as a categorical variable, with values ranging from 1943 to 1960.

Given that higher birth order is logically dependent on a larger family size, we included sibling group size to account for the total number of children born to the mother. Sibling group size was coded categorically as two to five children. We also included a measure of birth spacing, that is, the length of the interval between each birth. Accounting for differences in birth spacing controls for much of the remaining variability in birth order between families. A shorter subsequent birth interval has also been associated with increased risk of hospitalization attributable to use of alcohol or narcotics (Riordan et al., 2012). Birth spacing was measured in terms of birth density, calculated as the range in years between the last- and first-born children divided by the total number of children.

We included the mother's year of birth to account for cohort trends, applied using individual year dummies in the analysis, with values between 1905 and 1939. Finally, the mother's age at first birth was included as a covariate to account for the association between early childbearing and birth order; mother's selection into early childbearing is further associated with relative sociodemographic disadvantage in early life (Hobcraft & Kiernan, 2001). Mother's age at first birth was similarly applied as individual year dummies, with values ranging from 14 to 45.

### 2.4 Statistical analyses

#### 2.4.1 Cox proportional hazards regression

We used Cox proportional hazards regression to estimate hazards for birth order for hospitalization and death. Due to the established etiologic differences in the development of alcohol and narcotics use and related health outcomes over time, analyses were conducted separately for each outcome.

The assumption of proportionality is the central tenet in Cox regression. For each set of analyses, the assumptions were assessed graphically via log-log plots of survival, and with formal tests of proportionality using Schoenfeld residuals (Grambsch & Therneau, 1994). Global tests of this assumption for the initial analyses suggested evidence of non-proportionality by sex, with disproportionately higher hazards among males, relative to females, at the beginning and end of follow-up. To mitigate inaccuracies in the estimates resulting from non-proportional hazards, all analyses were subsequently stratified by sex (dichotomously coded according to biological sex at birth). Further, the global test of proportionality on the basis of Schoenfeld residuals for narcotics shows some evidence of non-proportionality (*p* = 0.0226), which is expected as narcotics use is subject to period effects (Giordano et al., 2014).

For comparisons between families, we fit a Cox model according to the following for individual *i*:

$$\lambda_{i}\left(t\right)=\lambda_{s\left[i\right]}\left(t\right)\;\exp\left(\beta_{1}{BO}_{i}+\beta_{2}{YOB}_{i}+\beta_{3}{SSIZE}_{i}+\beta_{4}{DEN}_{i}+\beta_{5}{MYOB}_{i}+\beta_{6}{MAGE}_{i}\right)\text{,}$$

where $\lambda_{i}\left(t\right)$ is the hazard for individual *i* at time *t*; $\lambda_{s\left[i\right]}\left(t\right)$is the baseline hazard for individual *i* of sex *s* at time *t*; ${BO}_{i}$ is birth order (reference category is the firstborn child); ${YOB}_{i}$ is the child's birth year; ${SSIZE}_{i}$ is the sibling group size; ${DEN}_{i}$ is the birth density; ${MYOB}_{i}$ is the mother's year of birth; and ${MAGE}_{i}$ is the mother's age at first birth. The timescale used for the baseline hazard was age for all analyses; individuals were followed from age 30 until hospitalization or death attributable to alcohol or narcotics use, and were right-censored at time of death attributable to another cause, emigration, or the end of follow-up (age 55). The results are presented as hazard ratios (HRs) and 95% confidence intervals (CIs).

To compare outcomes among siblings within the same family, we applied two fixed effects approaches via stratified Cox models. The family fixed effects approach accounts for unobserved, time-invariant characteristics within the family environment, which reduces confounding, and thereby bias, in the estimates (e.g., Barclay et al., 2016; Barclay & Kolk, 2015; Bjørngaard et al., 2013; Rostila et al., 2014). However, this approach requires variation in the outcome: at least one sibling must have had an event to be included in the sample (Allison, 2009). This restriction reduces our analytic populations to the families in which at least one sibling had a hospitalization or death event attributable to alcohol (n = 6223 families; n = 17,934 individuals) or narcotics (n = 2476 families; n = 7040 individuals), respectively. Here, we estimate birth order effects by comparing siblings within the same family. We use stratified Cox models, where the strata correspond to the shared sibling group ID, and take the following form:

$$\lambda_{i}\left(t\right)=\lambda_{j\left[i\right]s\left[i\right]}\left(t\right)\;\exp\left(\beta_{1}{BO}_{i}+\beta_{2}{YOB}_{i}\right)\text{,}$$

where $\lambda_{j\left[i\right]s\left[i\right]}\left(t\right)$ is the baseline hazard for individual *i* of sex *s* in family *j*. Time-invariant familial background factors drop out, but we still adjusted for the child's year of birth ${YOB}_{i}$ as this is not a factor shared between siblings.

Black et al. (2018) proposed an alternative approach, which compares differences in outcomes by birth order in families with the same number of children and children born in the same years. In other words, the variability in birth order is estimated within families of the same type (Black et al., 2018). Though this approach still balances family background factors by birth order, it does not account for all unobserved familial characteristics. Conversely, as this approach does not necessitate variation in the outcome, we retain the entire analytic population. We therefore applied Black et al. family type fixed effects approach by stratifying according to the combination of the sibling group size and all siblings' years of birth (Black et al., 2018), thereby allowing a shared baseline hazard among families of the same type (Allison, 2009). We estimated the following model:

$$\lambda_{i}\left(t\right)=\lambda_{f\left[i\right]s\left[i\right]}\left(t\right)\;\exp\left(\beta_{1}{BO}_{i}+\beta_{2}{YOB}_{i}\right)\text{,}$$

where $\lambda_{f\left[i\right]s\left[i\right]}\left(t\right)$ is the baseline hazard for individual *i* in family type *f* of sex *s*.

All statistical analyses were conducted using Stata version 16 (StataCorp, 2019).

## 3 Results

### 3.1 Descriptive statistics

Characteristics of the sample population are shown in Table 1. There were 5109 alcohol-attributable events (of which 3.1% were deaths) and 2110 events (3.7% deaths) attributable to use of narcotics, with a mean time at risk of 11.2 and 10.3 person-years, respectively, among those with events. For both outcomes, incidence rates (per 1000 person-years) increase slightly with both higher birth order and sibship size. The opposite pattern is observed for mother's age at first birth, with generally decreasing incidence rates with decreasing mother's age at first birth. The percentage of males is nearly three times as high as that of females for alcohol-attributable events, and over 1.5 times higher among males, relative to females, for narcotics-attributable events. For alcohol-attributable events, there is little variation among incidence rates with respect to birth year, whereas incidence rates for narcotics-attributable events are slightly higher among those born 1953 and later.

**Table 1:** Descriptive statistics for the study population, alcohol- and narcotics-attributable hospitalization or death (ages 30–55), by person-years.

	Alcohol			Narcotics		
	Person-years N (%)	Events N (%)	Incidence rate (95% CI) a	Person-years N (%)	Events N (%)	Incidence rate (95% CI) a
**Birth order**						
First	1,334,223 (37.8)	1850 (36.2)	1.39 (1.32–1.45)	1,347,990 (37.9)	654 (31.0)	0.49 (0.45–0.52)
Second	1,332,621 (37.8)	1843 (36.1)	1.38 (1.32–1.45)	1,343,749 (37.7)	818 (38.8)	0.61 (0.57–0.65)
Third	607,649 (17.2)	917 (17.9)	1.51 (1.41–1.61)	613,546 (17.2)	434 (20.6)	0.71 (0.64–0.78)
Fourth	200,603 (5.7)	385 (7.5)	1.92 (1.74–2.12)	203,144 (5.7)	164 (7.8)	0.81 (0.69–0.94)
Fifth	50,540 (1.4)	114 (2.2)	2.26 (1.88–2.71)	51,308 (1.4)	40 (1.9)	0.78 (0.57–1.06)
**Sex**						
Male	1,801,837 (51.1)	3781 (74.0)	2.10 (2.03–2.17)	1,830,319 (51.4)	1315 (62.3)	0.72 (0.68–0.76)
Female	1,723,799 (48.9)	1328 (26.0)	0.77 (0.73–0.81)	1,729,418 (48.6)	795 (37.7)	0.46 (0.43–0.49)
**Birth year**^b^						
<1953	1,277,819 (36.2)	1869 (36.6)	1.46 (1.40–1.53)	1,292,324 (36.3)	633 (30.0)	0.49 (0.45–0.53)
1953	1,335,033 (37.9)	1890 (37.0)	1.42 (1.35–1.48)	1,347,141 (37.8)	817 (38.7)	0.61 (0.57–0.65)
>1953	912,784 (25.9)	1350 (26.4)	1.48 (1.40–1.56)	920,272 (25.9)	660 (31.3)	0.72 (0.66–0.77)
**Sibling group size**						
Two	1,452,910 (41.2)	1791 (35.1)	1.23 (1.18–1.29)	1,463,902 (41.1)	746 (35.4)	0.51 (0.47–0.55)
Three	1,217,875 (34.5)	1770 (34.6)	1.45 (1.39–1.52)	1,229,801 (34.5)	770 (36.5)	0.63 (0.58–0.67)
Four	599,469 (17.0)	1026 (20.1)	1.71 (1.61–1.82)	606,433 (17.0)	415 (19.7)	0.68 (0.62–0.75)
Five	255,382 (7.2)	522 (10.2)	2.04 (1.88–2.23)	259,601 (7.3)	179 (8.5)	0.69 (0.60–0.80)
**Mother's age at 1**st **birth**^c^						
<20	406,374 (11.5)	892 (17.5)	2.20 (2.06–2.34)	412,404 (11.6)	391 (18.5)	0.95 (0.86–1.05)
20-24	1,475,324 (41.8)	2413 (47.2)	1.64 (1.57–1.70)	1,491,281 (41.9)	1000 (47.4)	0.67 (0.63–0.71)
25-29	1,127,863 (32.0)	1258 (24.6)	1.12 (1.06–1.18)	1,136,275 (31.9)	508 (24.1)	0.45 (0.41–0.49)
30-34	417,127 (11.8)	438 (8.6)	1.05 (0.96–1.15)	419,919 (11.8)	166 (7.9)	0.40 (0.34–0.46)
>34	98,949 (2.8)	108 (2.1)	1.09 (0.90–1.32)	99,858 (2.8)	45 (2.1)	0.45 (0.34–0.60)
**Total**	3,525,636 (100)	5109 (100)	1.45 (1.41–1.49)	3,559,737 (100)	2110 (100)	0.59 (0.57–0.62)

a Incidence rates are calculated per 1000 person-years.

b Included as individual year dummies in the analysis, but presented categorically here to illustrate the unique nature of the data material.

c Included as individual year dummies in the analysis, but presented in age bands for descriptive purposes.

### 3.2 Differences between families according to birth order for alcohol- and narcotics-attributable events

Table 2 shows the estimates from Cox regression analyses conducted between families. The bivariate results (Model 1) suggest a positive relationship between birth order and alcohol- and narcotics-attributable events; we generally observe increasing hazards with increasing birth order. In the analysis adjusted for the family background variables (Model 2), the contribution of birth order to the variability in alcohol-attributable hospitalization or death events is less evident, with HRs ranging from 0.92 to 1.08 and 95% CIs including one. For narcotics-attributable events, the positive relationship with birth order generally persists, though all 95% CIs also include one in the adjusted model.

**Table 2:** Estimates of alcohol- and narcotics-attributable hospitalization or death (ages 30–55) between families, according to birth order. Results from Cox regression analyses.

	Alcohol				Narcotics			
	Model 1		Model 2 a		Model 1		Model 2 a	
	HR	95% CI	HR	95% CI	HR	95% CI	HR	95% CI
**Birth order**								
Second	1.00	0.94–1.07	0.96	0.87–1.06	1.26***	1.13–1.39	1.13	0.96–1.32
Third	1.10*	1.01–1.19	0.92	0.78–1.09	1.46***	1.30–1.65	1.15	0.89–1.49
Fourth	1.40***	1.25–1.56	1.04	0.82–1.31	1.67***	1.41–1.98	1.24	0.86–1.79
Fifth	1.63***	1.35–1.97	1.08	0.78–1.50	1.61**	1.17–2.21	1.18	0.70–2.01
**N**	147,952				147,952			
**Events (N)**	5109				2110			

Notes: The sample is restricted to families of two to five children in which all children were born 1943–1960. Reference category is the first-born child. All models are stratified by sex.

HR=Hazard Ratio; CI=Confidence interval; statistical significance: *p < 0.05; **p < 0.01; ***p < 0.001.

a Includes dummy variables for the child's year of birth, mother's year of birth, mother's age at first birth, and sibling group size; continuous measure of birth density; and multiplicative interaction term between sibling group size and birth density.

### 3.3 Birth order differences in fixed effects models for alcohol- and narcotics-attributable events

The results of the family fixed effects approach are presented in Table 3. The estimates suggest higher hazards for both alcohol- and narcotics-attributable events among second- through fifth-born children, relative to first-borns, though a clearly graded association is not observed. However, the 95% CIs largely overlap between birth order categories and all 95% CIs in the models include one.

**Table 3:** Estimates of alcohol- and narcotics-attributable hospitalization or death (ages 30–55), according to birth order and including family fixed effects. Results from Cox regression analyses.

	Alcohol		Narcotics	
	Model 1 a		Model 1 a	
	HR	95% CI	HR	95% CI
**Birth order**				
Second	1.06	0.91–1.24	1.15	0.90–1.46
Third	1.02	0.80–1.30	1.09	0.74–1.61
Fourth	1.19	0.85–1.68	1.05	0.61–1.81
Fifth	1.30	0.82–2.05	1.13	0.53–2.40
**N**	17,934		7040	
**Events (N)**	5109		2110	

Notes: The sample is restricted to families of two to five children in which all children were born 1943–1960 and in sibling groups with variance on the outcome variable. Reference category is the first-born child. All models are stratified by sex.

HR=Hazard Ratio; CI=Confidence interval; statistical significance: *p < 0.05; **p < 0.01; ***p < 0.001.

a Stratified by sibling group, and includes dummy variables for child's year of birth.

Results from the analyses applying family type fixed effects are shown in Table 4. We generally observe a positive association between birth order and alcohol- and narcotics-attributable events, where second-through fifth-borns have a higher hazard for hospitalization or death attributable to use of alcohol or narcotics, relative to first-borns. As in Table 3, the 95% CIs generally overlap between second- and higher-order births, and all 95% CIs for both outcomes include one.

**Table 4:** Estimates of alcohol- and narcotics-attributable hospitalization or death (ages 30–55), according to birth order and including family type fixed effects. Results from Cox regression analyses.

	Alcohol		Narcotics	
	Model 1 a		Model 1 a	
	HR	95% CI	HR	95% CI
**Birth order**				
Second	1.05	0.94–1.19	1.12	0.93–1.36
Third	1.08	0.89–1.32	1.18	0.85–1.62
Fourth	1.29	0.98–1.69	1.23	0.79–1.93
Fifth	1.46	1.00–2.13	1.22	0.65–2.30
**N**	147,952		147,952	
**Events (N)**	5109		2110	

Notes: The sample is restricted to families of two to five children in which all children were born 1943–1960.

Reference category is the first-born child. All models are stratified by sex.

HR=Hazard Ratio; CI=Confidence interval; statistical significance: *p < 0.05; **p < 0.01; ***p < 0.001.

a Stratified by family type, and includes dummy variables for child's year of birth.

### 3.4 Sensitivity analyses

Given that higher birth order is logically dependent on a larger family size, we estimate birth order differences between families by sibling group size for each outcome. These results are presented in Tables A1 and A2 for alcohol and narcotics, respectively. The sibling group size-specific results in Table A1 show a similar pattern as the adjusted results presented in Table 2: second- and third-borns have slightly lower hazards relative to first-borns, whereas fourth- and fifth-borns have slightly higher hazards for alcohol-attributable events. In families with two siblings, the most common family size in Sweden (Andersson, 1999), we observe slightly higher hazards among second-borns, relative to first-borns. The results presented in Table A2 also generally show the same patterns as the adjusted model presented in Table 2; relative to first-borns, all later-borns have higher hazards for a narcotics-attributable event. The exception is among families with two children, where the hazards for first- and second-born children are identical. Importantly, however, 95% CIs include one for all results according to sibling group size for both outcomes.

The data material is uniquely derived around the 1953 birth cohort. Though the same follow-up period (ages 30–55) is used for the entire analytic population, the 17-year difference between the oldest (1943) and youngest (1960) birth cohorts could introduce unmeasured cohort effects into the analysis. We therefore estimated birth order differences between families using only the index cohort sample (n = 55,928 egos) to minimize cohort effects; the results are presented in Table A3. The bivariate results show the same positive relationship between birth order and both outcomes as seen in Table 2. The adjusted models also do not suggest clear birth order differences for alcohol-attributable events, with similar hazards for first- through fourth-born children, and slightly higher hazards among fifth-borns. For narcotics-attributable events, we observe higher hazards among second- and third-born children, and slightly lower hazards among fourth- and fifth-borns, relative to first-borns, in the adjusted analysis. All 95% CIs in the adjusted models include one for both outcomes.

Finally, some misclassification of sibling group size, and therefore birth order, could have resulted from the reduced coverage of deceased individuals in the Multigenerational Register between 1968 and 1991 (Statistics Sweden, 2017). We conducted several simulations with realistic parameters for mortality rates to determine whether any such misclassification may have impacted our estimates. The simulations (results not shown) indicated that the selection bias introduced by the reduced coverage during this period was unlikely to have a significant influence on the presented results.

## 4 Discussion

Prior research on birth order indicates that later-born children have a higher risk for adverse mental and behavioral health outcomes, relative to their earlier-born siblings, before reaching young adulthood. This study adds a unique contribution to the literature by using high-quality Swedish register data over a 25-year period to estimate the effects of birth order between and within families on health outcomes attributable to use of alcohol or narcotics during midlife, a discrete age period for which birth order effects have not been estimated.

Here, and in other birth order studies, the aim was to estimate the direct effects of birth order, or the variability in alcohol- or narcotics-attributable events by birth order. In comparisons between families, our unadjusted results suggest a positive relationship between birth order and alcohol- and narcotics-attributable events, wherein we generally observe increasing hazards with increasing birth order. However, the direct effects of birth order were reduced when holding familial background factors constant. The results from both fixed effects approaches show similarly higher hazards among higher birth order individuals, relative to first-borns, but the 95% CIs largely overlap between higher-order births when adjusting for time-invariant family and family type characteristics, respectively. Our findings therefore suggest that, for these outcomes, the previously observed birth order differences prior to young adulthood may be less evident in later life course stages, and differences between siblings in midlife can, to a large degree, be explained by other variables within the family of origin.

Aligning with a prior study that uses contemporary Swedish register data and family fixed effects to estimate birth order effects on these outcomes (Barclay et al., 2016), we explicitly and implicitly adjust for time-invariant familial background factors that could have long-term implications for all children within the sibling group; these factors are one potential mechanism underlying the limited direct effects of birth order in midlife. In our comparisons between families, the hazards among individuals in larger sibling groups with shorter birth intervals were between 45% and over two times higher than those from families of two children (these results are presented in Table A4). Selection into larger family size is one possible explanation for the higher hazards among later-born children despite the diminished effects after adjustment for family background characteristics. Disadvantaged socioeconomic circumstances are associated with higher completed parity (Jalovaara et al., 2022) and shorter birth intervals (Winikoff, 1983). Higher birth density and a larger family size may further limit the allocation of economic resources available per child (Powell & Steelman, 1995). Similar selection mechanisms could explain the results found in the fixed effects analyses. Mother's age at first birth is a time-invariant characteristic shared across the sibling group, and is therefore implicitly adjusted for in these analyses. Mothers who select into early childbearing are correspondingly more likely to come from disadvantaged backgrounds themselves (Kiernan, 1997). Family background characteristics, including disadvantaged socioeconomic circumstances, are established, long-term predictors of alcohol- and narcotics-attributable events among offspring, even into adulthood (Barr et al., 2018; Osler et al., 2006). These factors may have long-term implications for all siblings, and thereby limit the direct effects of birth order in midlife.

Our results highlight the importance of considering the social environment in a given period in the life course. Social influence is one theoretical mechanism attributed to birth order differences in the uptake and continuity of alcohol or narcotics use within sibling groups (Whiteman et al., 2013). Our follow-up period occurs after offspring have typically left the parental home and established lives independent of their shared families. As could be expected, the direct effects of birth order seem limited in adulthood when the influences of social relationships within the current family constellation, such as cohabitating partners, spouses, or live-in children, may have a stronger contribution to the transmission of risk and protective health behaviors (Borschmann et al., 2019; Duncan et al., 2006; Kendler et al., 2019; Salvatore et al., 2020).

Finally, these results underscore the potentially dynamic influence of familial characteristics on alcohol and narcotics use across the life course. Though direct effects of birth order may be more evident at younger ages, our results suggest that familial background factors beyond that of birth order may contribute to adverse health outcomes among siblings, which may include both environmental and genetic factors. Substantial evidence shows that there are significant environmental and genetic influences on the development and transmission of substance use disorders (Kendler et al., 2016, 2021). Further, these influences may have differential effects for the development and transmission of substance use disorders during discrete life course stages (Long et al., 2017). Future research is needed to provide additional insights into the potentially time-inconstant contributions of family background and these outcomes throughout the life course.

### 4.1 Limitations

This study uses unique data material to estimate associations between birth order and hospitalization or death attributable to use of alcohol or narcotics for a national Swedish birth cohort and their siblings for 25 years during the same stage of the life course (ages 30 to 55). This study is based on high-quality data and we use appropriate methods to address our research aim; however, it is not without limitations.

The limited evidence of direct birth order effects on the outcomes could partly be a result of the observation period under study. It is possible that direct birth order effects could have been observed for this population had we been able to measure hospitalizations occurring before age 30 for all siblings and subsequently compare observed birth order differences between this age period and midlife. However, rather than the acute intoxication often reflected in adolescent diagnoses, our measure of first hospitalization or death likely captures substance use-related disorders and diseases that mainly affect adults. As morbidity and mortality attributable to substance use are increasing among middle and older aged individuals (Kelly & Vuolo, 2021; Lehmann & Fingerhood, 2018; Yarnell et al., 2020), disentangling the contribution of family background factors for these outcomes in midlife is of increasing importance.

The higher hazards generally observed among individuals of higher birth order may partly reflect the sample. As birth order was based on the mother's full fertility history, sibling groups may include children of differing paternity (half-siblings). The commonality of familial complexity in Sweden has been increasing since the 1950s (Thomson, 2014), and prior research suggests that birth order patterns within families may be better explained by the social, rather than the biological, order within the sibling group (Barclay, 2015; Black et al., 2018). As familial instability is also associated with alcohol and drug use disorders in adulthood (Kendler et al., 2002; Osler et al., 2006), the increased hazards among higher birth order individuals may reflect more complex family constellations.

Despite the high validity of diagnoses in the National Patient Register (Ludvigsson et al., 2011), it is plausible that some measurement error was introduced due to misclassification, inconsistencies in diagnosing inpatient events between doctors or care facilities, or changes in the diagnostic criteria for alcohol- or narcotics-attributable events between the eighth and tenth revisions of the ICD. National coverage of inpatient register data is available from 1987, though coverage was considered complete in Sweden's three largest counties (approximately 50% of the population at the time) since 1973, the earliest observation period in this study. However, this incomplete coverage may have resulted in some underestimation among the earlier cohorts. Our inclusion of both the underlying and secondary causes of hospitalization aimed to ameliorate some of these potential sources of error. Our outcome variables are further based on hospitalization or death, and therefore only include severe events or events for which individuals sought treatment. Given the 25-year follow-up period in our study, many individuals who continuously use substances are likely treated by the Swedish healthcare system; however, it is difficult to determine the generalizability of our results to other studies in which other harmful patterns of use not resulting in inpatient care or death are included.

The generalizability of our findings to contemporaneous Swedish cohorts, or cohorts in other contexts, may be limited by the data material. There are 17 birth years represented among the sibling cohorts, which could result in unmeasured cohort effects. However, restricting these samples to include families where all children were born between 1943 and 1960, and thereby allowing for the same age at entry and exit, aims to mitigate this issue. Further, given our sensitivity analysis using the single birth cohort yielded similar patterns by birth order, the variation in risk for these outcomes is unlikely to be purely the result of cohort effects. Finally, distinctive age, period, and cohort trends have been observed regarding hospitalization or death due to use of alcohol and narcotics in Sweden during our observation period (Giordano et al., 2014; Rosén & Haglund, 2019), which may result in different rates of hospitalization between the birth cohorts and unmeasured differences in who is hospitalized over time. However, use of a nationally-representative cohort and their siblings with an extended follow-up time is a distinct strength in helping to disentangle the mechanisms underlying the associations between social and environmental factors in early life and adverse adult health outcomes.

### 4.2 Conclusions

In summary, our study finds limited evidence for the direct effects of birth order for both outcomes in midlife. These results further support the idea that the risks for adverse mental and behavioral health events are dynamic, and familial characteristics may differentially influence these outcomes during different life course stages. Future research should further disentangle the time-varying contributions of background variables in the family of origin, including family instability and complexity, for birth order differences in alcohol- and narcotics-attributable events among siblings during discrete periods in the life course.

## Author contributions

Contributors: LB and KB planned and designed the study. KB advised on methodology. LB analyzed the data, and LB and KB interpreted the results. LB drafted the original manuscript; LB and KB reviewed and revised the manuscript, and approved the final manuscript for submission. LB attests that all listed authors meet authorship criteria and that no others meeting the criteria have been omitted.

## Declarations of competing interest

None.

## Data Availability

The authors do not have permission to share data.
