# Supplementary material for: Birth order and health events attributable to alcohol and narcotics in midlife: A 25-year follow-up of a national Swedish birth cohort and their siblings
Source: SSM Popul Health. 2022 Aug 28;19:101219. doi: 10.1016/j.ssmph.2022.101219 (PMC9450127; doi:10.1016/j.ssmph.2022.101219)
Supplement: Multimedia component 1 [file mmc1.docx]

**Table A1: Estimates of alcohol-attributable hospitalization or death (ages 30-55) between families, according to birth order, by sibling group size. Results from Cox regression analyses.**

|  |  | **Sibship size** | | |  | | |  | | |  | |
| --- | --- | --- | --- | --- | --- | --- | --- | --- | --- | --- | --- | --- |
|  |  | **2** | | | **3** | | | **4** | | | **5** | |
|  |  | Model 1 ^a^ | | | Model 2 ^a^ | | | Model 3 ^a^ | | | Model 4 ^a^ | |
|  |  | HR | 95% CI |  | HR | 95% CI |  | HR | 95% CI |  | HR | 95% CI |
| **Birth order** |  |  |  |  |  |  |  |  |  |  |  |  |
| Second |  | 1.14 | 0.89–1.46 |  | 0.89 | 0.75–1.05 |  | 0.96 | 0.78–1.18 |  | 0.79 | 0.57–1.09 |
| Third |  |  |  |  | 0.85 | 0.64–1.14 |  | 0.82 | 0.60–1.10 |  | 0.91 | 0.61–1.37 |
| Fourth |  |  |  |  |  |  |  | 0.85 | 0.56–1.29 |  | 1.09 | 0.64–1.84 |
| Fifth |  |  |  |  |  |  |  |  |  |  | 1.16 | 0.58–2.30 |
| **N** |  | 60,737 |  |  | 51,194 |  |  | 25,240 |  |  | 10,781 |  |
| **Hospitalizations (N)** |  | 1,791 |  |  | 1,770 |  |  | 1,026 |  |  | 522 |  |

Notes: The sample is restricted to families of two to five children in which all children were born 1943-1960. Each column represents a separate regression. Reference category is the firstborn child. All models are stratified by sex.

^a^ Includes dummy variables for the child’s year of birth, mother’s year of birth, and mother’s age at first birth; and continuous measure of birth density.

HR=Hazard Ratio; CI=Confidence interval; statistical significance: * *p* < 0.05; ** *p* < 0.01; *** *p* < 0.001

**Table A2: Estimates of narcotics-attributable hospitalization or death (ages 30-55) between families, according to birth order, by sibling group size. Results from Cox regression analyses.**

|  |  | **Sibship size** | | |  | | |  | | |  | |
| --- | --- | --- | --- | --- | --- | --- | --- | --- | --- | --- | --- | --- |
|  |  | **2** | | | **3** | | | **4** | | | **5** | |
|  |  | Model 1 ^a^ | | | Model 2 ^a^ | | | Model 3 ^a^ | | | Model 4 ^a^ | |
|  |  | HR | 95% CI |  | HR | 95% CI |  | HR | 95% CI |  | HR | 95% CI |
| **Birth order** |  |  |  |  |  |  |  |  |  |  |  |  |
| Second |  | 1.00 | 0.69–1.45 |  | 1.13 | 0.87–1.48 |  | 1.24 | 0.89–1.74 |  | 1.02 | 0.56–1.87 |
| Third |  |  |  |  | 1.34 | 0.86–2.07 |  | 1.03 | 0.64–1.66 |  | 1.32 | 0.64–2.72 |
| Fourth |  |  |  |  |  |  |  | 1.15 | 0.59–2.23 |  | 1.67 | 0.68–4.11 |
| Fifth |  |  |  |  |  |  |  |  |  |  | 1.44 | 0.44–4.65 |
| **N** |  | 60,737 |  |  | 51,194 |  |  | 25,240 |  |  | 10,781 |  |
| **Hospitalizations (N)** |  | 746 |  |  | 770 |  |  | 415 |  |  | 179 |  |

Notes: The sample is restricted to families of two to five children in which all children were born 1943-1960. Each column represents a separate regression. Reference category is the firstborn child. All models are stratified by sex.

^a^ Includes dummy variables for the child’s year of birth, mother’s year of birth, and mother’s age at first birth; and continuous measure of birth density.

HR=Hazard Ratio; CI=Confidence interval; statistical significance: * *p* < 0.05; ** *p* < 0.01; *** *p* < 0.001

**Table A3: Estimates of alcohol- and narcotics-attributable hospitalization or death (ages 30-55) between families, according to birth order. Results from Cox regression analyses.**

|  |  | **Alcohol** | | | | | | | | |  | **Narcotics** | | | | |  |
| --- | --- | --- | --- | --- | --- | --- | --- | --- | --- | --- | --- | --- | --- | --- | --- | --- | --- |
|  |  | Model 1 | | |  | | Model 2 ^a^ | | | |  | Model 1 | |  | Model 2 ^a^ | |  |
|  |  | HR | | 95% CI | |  | | HR | | 95% CI |  | HR | 95% CI |  | HR | 95% CI |  |
| **Birth order** |  |  | |  | |  | |  | |  |  |  |  |  |  |  |  |
| Second |  | 1.08 | | 0.97–1.20 | |  | | 1.04 | | 0.88–1.24 |  | 1.22* | 1.04–1.44 |  | 1.17 | 0.90–1.52 |  |
| Third |  | 1.23** | | 1.07–1.40 | |  | | 1.04 | | 0.79–1.36 |  | 1.33** | 1.09–1.63 |  | 1.05 | 0.70–1.59 |  |
| Fourth |  | 1.50*** | | 1.22–1.83 | |  | | 1.06 | | 0.73–1.56 |  | 1.44* | 1.04–1.99 |  | 0.88 | 0.50–1.56 |  |
| Fifth |  | 2.04** | | 1.33–3.12 | |  | | 1.15 | | 0.63–2.09 |  | 2.03* | 1.04–3.95 |  | 0.96 | 0.39–2.39 |  |
| **N** |  | 55,928 |  | |  | |  | | |  |  | 55,928 |  |  |  |  |  |
| **Events (N)** |  | 1,890 |  | |  | |  | |  | |  | 817 |  |  |  |  |  |
| Notes: The sample is restricted to the 1953 index persons born to families of two to five children in which all children were born 1943-1960. Reference category is the firstborn child. All models are stratified by sex.  ^a^ Includes dummy variables for the mother’s year of birth, mother’s age at first birth, and sibling group size; continuous measure of birth density; and multiplicative interaction term between sibling group size and birth density.  HR=Hazard Ratio; CI=Confidence interval; statistical significance: * *p* < 0.05; ** *p* < 0.01; *** *p* < 0.001 | | | | | | | | | | | | | | | | |  |

**Table A4: Estimates of alcohol- and narcotics-attributable hospitalization or death (ages 30-55) between families, according to birth order. Results from Cox regression analyses.**

|  |  | **Alcohol** | | | | |  | **Narcotics** | | | | |
| --- | --- | --- | --- | --- | --- | --- | --- | --- | --- | --- | --- | --- |
|  |  | Model 1 | |  | Model 2 ^a^ | |  | Model 1 | |  | Model 2 ^a^ | |
|  |  | HR | 95% CI |  | HR | 95% CI |  | HR | 95% CI |  | HR | 95% CI |
| **Birth order** |  |  |  |  |  |  |  |  |  |  |  |  |
| Second |  | 1.00 | 0.94–1.07 |  | 0.96 | 0.87–1.06 |  | 1.26*** | 1.13–1.39 |  | 1.13 | 0.96–1.32 |
| Third |  | 1.10* | 1.01–1.19 |  | 0.92 | 0.78–1.09 |  | 1.46*** | 1.30–1.65 |  | 1.15 | 0.89–1.49 |
| Fourth |  | 1.40*** | 1.25–1.56 |  | 1.04 | 0.82–1.31 |  | 1.67*** | 1.41–1.98 |  | 1.24 | 0.86–1.79 |
| Fifth |  | 1.63*** | 1.35–1.97 |  | 1.08 | 0.78–1.50 |  | 1.61** | 1.17–2.21 |  | 1.18 | 0.70–2.01 |
| **Sibling group size** |  |  |  |  |  |  |  |  |  |  |  |  |
| Two |  |  |  |  | 1.00 |  |  |  |  |  | 1.00 |  |
| Three |  |  |  |  | 0.92 | 0.78–1.08 |  |  |  |  | 0.99 | 0.77–1.28 |
| Four |  |  |  |  | 0.75* | 0.60–0.95 |  |  |  |  | 0.81 | 0.57–1.17 |
| Five |  |  |  |  | 0.78 | 0.55–1.10 |  |  |  |  | 0.82 | 0.46–1.48 |
| **Birth density** |  |  |  |  | 1.00 | 0.86–1.16 |  |  |  |  | 1.12 | 0.89–1.41 |
| **Interaction**^b^ |  |  |  |  |  |  |  |  |  |  |  |  |
| Two |  |  |  |  | 1.00 |  |  |  |  |  | 1.00 |  |
| Three |  |  |  |  | 1.45** | 1.10–1.91 |  |  |  |  | 1.33 | 0.88–2.01 |
| Four |  |  |  |  | 2.54*** | 1.71–3.77 |  |  |  |  | 2.08* | 1.13–3.84 |
| Five |  |  |  |  | 2.76** | 1.53–4.95 |  |  |  |  | 1.84 | 0.69–4.95 |
| **N** |  | 147,952 |  |  |  |  |  | 147,952 |  |  |  |  |
| **Events (N)** |  | 5,109 |  |  |  |  |  | 2,110 |  |  |  |  |
| Notes: The sample is restricted to families of two to five children in which all children were born 1943-1960. Reference categories is the firstborn child. All models are stratified by sex.  ^a^ Includes dummy variables for the child’s year of birth, mother’s year of birth, and mother’s age at first birth.  ^a^ Refers to the multiplicative interaction term between sibling group size and birth density.  HR=Hazard Ratio; CI=Confidence interval; statistical significance: * *p* < 0.05; ** *p* < 0.01; *** *p* < 0.001 | | | | | | | | | | | | |
